# Validating Measures of Electrodermal Activity and Heart Rate Variability Derived From the Empatica E4 Utilized in Research Settings That Involve Interactive Dyadic States

**DOI:** 10.3389/fnbeh.2020.00148

**Published:** 2020-08-18

**Authors:** Nir Milstein, Ilanit Gordon

**Affiliations:** ^1^Department of Psychology, Bar-Ilan University, Ramat Gan, Israel; ^2^The Gonda Multidisciplinary Brain Research Center, Bar-Ilan University, Ramat Gan, Israel

**Keywords:** Empatica E4, MindWare mobile impedance cardiograph, interbeat intervals, heart rate variability, electro-dermal activity, dyadic research, heart rate

## Abstract

Portable and wireless devices that collect physiological data are becoming more and more sought after in clinical and psychophysiological research as technology swiftly advances. These devices allow for data collection in interactive states, such as dyadic therapy, with reduced restraints compared to traditional laboratory devices. One such portable device is the Empatica E4 wristband (Empatica Srl, Milan, Italy) which allows quantifying cardiac interbeat intervals (IBIs), heart rate variability (HRV), and electro-dermal activity (EDA), as well as several other acceleration and temperature measures. In the current study, we aimed to assess IBI, HRV, and EDA measures, against the same data collected from the well-validated MindWare mobile impedance cardiograph device (MindWare Technology, Gahanna, OH, United States). We assessed the E4 strictly as a research instrument and not as a clinical tool. We were specifically interested in the wristbands’ performance during naturalistic interactive face-to-face conversations which inherently involve more hand movements. We collected data from 30 participants, nested in 15 dyads, which were connected to both devices simultaneously, during rest and during a social conversation. After preprocessing and analyses, we found that mean IBIs obtained by the E4 and the MindWare device, were highly similar during rest and during conversation. Medium to high correlations were found between the devices with respect to several HRV measures, with higher correlations during rest compared to conversation. The E4 failed to produce reliable EDA data. We conclude by discussing the strengths and limitations of the E4 during seated conversational states and suggest optimal ways to collect and analyze data with the E4.

## Introduction

Acquiring psychophysiological data during naturalistic interactive states is of great interest to many psychological researchers (Morris and Aguilera, 2012), especially for clinical and socially focused research, due to higher external validity compared to data collection from individuals undergoing confining laboratory procedures. Laboratory psychophysiological experiments sometimes present a biased picture of the social phenomenon examined (Zaki and Ochsner, 2009; Shamay-Tsoory and Mendelsohn, 2019), as the setting as well as restraints on movement may lead to non-naturalistic situations (e.g., participants are connected to multiple electrodes and wires, the experiment must take place in the laboratory). These restrictions might result in limited ecological validity (Raugh et al., 2019). Many researchers avoid measuring psychophysiological data due to these reasons and others, such as high costs of well-validated systems for acquiring and analyzing data. Addressing these issues, portable wearable devices, which allow for more naturalistic physiological data collection during dynamic interactive settings, are becoming more and more sought after in psychological research (Morris and Aguilera, 2012; Vilardaga et al., 2014). However, independent validation research for these devices is scarce. It is important to have an independent validation for these devices, in order to inform their use in behavioral research.

The current study is seeking to evaluate one such device - the Empatica E4 wristband (Empatica Srl, Milan, Italy), specifically during interactive states. We aimed to assess the E4 strictly as a research instrument and not as a clinical tool. The Empatica E4 wristband is a wearable, non-invasive, research device which allows for real-time physiological data collection such as blood volume pulse (BVP) from which heart rate (HR), interbeat intervals (IBIs) in milliseconds and heart rate variability (HRV) is derived. It also allows collecting electro-dermal activity (EDA), body temperature, and acceleration. One of the great advantages of the E4 wristband is that it can be easily deployable outside the lab whereas this is more challenging with more traditional and established systems, such as BIOPAC (BIOPAC Systems Inc., Goleta, CA, United States) and MindWare (MindWare Technology, Gahanna, OH, United States). As noted above, the E4 wristband also involves fewer restrictions on natural movements of the participant.

For the purposes of this specific paper, we will focus on three extensively studied measures of activity of the autonomic nervous system – IBI, HRV, and EDA. It is advantageous to assess IBI, HRV, and EDA, since these measures capture both parasympathetic and sympathetic components of the nervous system (see Blascovich et al., 2011). As measuring HRV does not require particularly invasive testing, it is extensively acquired in psychological science and is considered a reliable estimate of autonomic nervous system modulation, via the vagus nerve, of sinus node cardiac activity (Blascovich et al., 2011; Kaufmann et al., 2011; Shaffer et al., 2014). From the IBI data it is possible to calculate HRV time domain measures such as Root Mean of Successive Differences (RMSSD) and Standard Deviation of Normal-to-Normal (SDNN). HRV frequency domain measures can also be produced based on the IBI series such as low frequency (LF) HRV and high frequency (HF) HRV spectral power. The HF band is also referred to as respiratory sinus arrhythmia (RSA) band due to the influence in this range of respiration on IBI variance. This frequency band in particular has been shown to represent parasympathetic nervous system regulation almost exclusively, and thus is a highly sought after (Berntson et al., 1993). RMSSD is also considered an index of the parasympathetic nervous system, while EDA is considered an index of the sympathetic nervous system. SDNN, which is a measure of general variability, HF, which represents cardiovagal activity (Camm et al., 1996) and IBI include both parasympathetic and sympathetic influences (Berntson et al., 2008). These measures can then be allow for an integrative assessment of differential influences of the sympathetic and parasympathetic branches of the autonomic nervous system on our periphery, allow us to quantify regulatory capacities (Berntson et al., 2008) and give us insight into dynamical changes in arousal as they occur during live interactive states, such as psychotherapy or other dyadic settings. One of the major advantages of the E4 is that it can measure both BVP (from which IBI and HRV are derived) and EDA. However, validation studies for these capabilities are scant.

Assessment of the E4 may not only indicate the absolute precision of the device, but can also indicate how well the E4 enables detection of physiological changes across different situations (relative precision). Physiological changes between different situations are of interest of many psychophysiological studies. For example, when tourists were asked to wear the E4 during their visit in Jerusalem, it was shown that EDA levels of visitors were higher when visiting historic parts of the city, compared to visiting the modern parts (Shoval et al., 2018).

The few validation studies of the Empatica E4 were presented in academic conferences, most of them did not assess IBI, HRV, and EDA measures or did not look for correlations between raw time-series (McCarthy et al., 2016; Ollander et al., 2017; Pietilä et al., 2017; Ragot et al., 2018). Thus, one advantage of the current investigation is measuring IBI, HRV, and EDA in a single design, which gives us indices of both branches of the nervous system, as well as the interaction between them. A second major advantage is the examination of correlations between raw time-series rather than only assessing correlations between mean estimations. Examining and visualizing pairs of time-series, collected by both devices simultaneously, allows us to evaluate the E4 more accurately. An exception is a more recent study (Menghini et al., 2019) which focused on stress reactions captured by the E4, while the current study focuses on natural dyadic interactive states.

It is important to assess interactive conversational states beyond the stress response, as they involve different and perhaps more subtle regulatory capacities of the autonomic nervous system (Porges, 2001), which are of specific interest to many researchers aiming to understand the mechanism underlying changes in HRV and EDA measures. Additionally, during conversation there are respiratory changes that can impact HRV measures (Quintana and Heathers, 2014), but most ambulatory measures cannot assess respiration in order to control for this potential artifact. Thus, it is especially important to assess the E4 performance while speaking. A final reason to include a conversation paradigm is the fact that more movement related artifacts occur during speaking, due to gesturing (Duncan, 1972).

Up to now, validation studies have found that the mean IBI data obtained by the E4 is accurate, especially during rest (Ollander et al., 2017; Pietilä et al., 2017; Menghini et al., 2019), and that it is less accurate for HRV (Ollander et al., 2017; Ragot et al., 2018; Menghini et al., 2019), again with higher accuracy during rest (Ollander et al., 2017; Pietilä et al., 2017; Menghini et al., 2019). For example, Menghini et al. (2019) report on no correlation between HRV measures derived by the E4 and the same HRV measures derived from the gold standard device during slow walking or keyboard typing (Pearson correlations ranged between 0.00 to 0.07). In contrast, they found nearly perfect correlations between HRV measures during rest (Pearson correlations ranged between 0.97 to 0.98). Three reported studies asked to validate the EDA data obtained by the E4 and found no resemblance between the EDA data obtained by the E4 and by a laboratory device (Ollander et al., 2017; Ragot et al., 2018; Menghini et al., 2019).

In light of the above, the goal of the current study was to evaluate IBI, HRV (both time and frequency domains) and EDA data collected by the Empatica E4 wristband, against the same measures derived from the well validated MindWare mobile impedance cardiograph (henceforth: MW mobile device) during socially meaningful interactive states. In order to achieve this aim, we performed a controlled study in which we measured IBI, HRV, and EDA, during rest and during a dyadic conversation, simultaneously by both the E4 and the MW mobile device. Our goal was to assess the accuracy of the E4 in collection of IBI, HRV, and EDA data during social states, by comparing this data to same physiological data obtained, in the same time, by the MW mobile device.

It should be noted that the two devices evaluated in this study collect cardiac signals differently. As mentioned above, the E4 uses BVP to assess cardiac outputs, while MW mobile device use direct electrical electrocardiograph (ECG) signal. Both techniques have pros and cons. The BVP, which is based on the volume of blood that passes through the tissues assessed via a photoplethysmogram (PPG) sensor, is easier to apply but is less specific than the ECG and subject to measurement errors. The electrode derived ECG is less sensitive to movement and considered more accurate. Thus, interactions, such as social conversations, should not greatly affect the quality of the data collected by WM mobile devices during such interactions. However, deriving HRV measures from the ECG technique is considered more complicated to perform and requires specialized software or expertise.

Although both devices acquire cardiac outputs differently, it seems that in some conditions (i.e., rest) the data extracted by the E4 is highly similar to the cardiac outputs extracted by ECG based devices, as suggested by preliminary investigations (Ollander et al., 2017; Pietilä et al., 2017).

Based on the findings of previous validation studies (McCarthy et al., 2016; Ollander et al., 2017; Pietilä et al., 2017; Ragot et al., 2018; Menghini et al., 2019), we hypothesize that the E4 will show higher resemblance to the MW mobile device during rest in comparison to a social interaction (dyadic conversation) since the photoplethysmogram sensor might be more sensitive to movement than ECG data collected by the MW mobile device via electrodes placed on the torso (Tamura et al., 2014; Pietilä et al., 2017). We also speculated, based on previous findings, that the IBI means estimated by the E4 will be more accurate than the HRV and the EDA estimations. We expected HRV data to be less accurate due to the fact that missing data impacts the estimation of HRV measures, which depend on accurately collecting the *entire* IBI time-series, much more compared to its effect on IBI mean estimation which can be more easily estimated based on average IBIs pre and post missing data.

## Materials and Methods

### Participants

Thirty healthy psychology undergraduate students (22 women and 8 men; *M* age = 26.28 years, *SD* = 6.05 years) participated in the study. They were randomly assigned to 15 dyads (eight dyads comprised of two women, six dyads comprised of a man and a woman and one dyad comprised of two men). Sample size was determined *a priori* via GPower 3.1.9.4 Software – considering we expected quite high effect sizes (correlations of *r* = 0.80), a sample size of 11 groups was recommended, which was estimated to yield a power of 0.95. To account for the fact that some effect sizes will be somewhat higher, and some smaller, we chose to include 15 groups in the study, with 30 individuals nested within them. All participants provided formal written informed consent. All participants reported that they were not diagnosed with cardiovascular diseases. The study was approved by the Departmental Ethical Committee.

### Tools

#### Mindware Mobile Impedance Cardiograph Device

The MW mobile device enables several channels of data collection, including ECG from which HR, HRV, and IBI can be derived, cardiograph impedance, EDA, acceleration, respiration and electromyography (EMG), in a sampling rate of 500 Hz. The MW mobile device measures ECG by a modified lead II configuration and with two MW electrodes placed on the bottom left rib and on the right collar bone (clavicle). Then, HRV data is derived by MW Technology’s HRV application software. The MW mobile device collects EDA from two Ag/AgCl MW electrodes placed on the non-dominant palm. Then, EDA data is derived from MW Technology’s EDA application software.

The analysis of the ECG signal obtained from the MW mobile device was performed using MW Technology’s HRV application software, version 3.1.3. The application includes artifact detection algorithms which detect irregular peaks in the IBI series. From the IBI series, HRV measures are derived. In the current study, a visual inspection and manual editing of the IBI data was completed by a trained graduate student to ensure proper removal of artifacts and ectopic beats (Be). The HRV application marks every irregular peak in the IBI series, which did not pass artifact detection algorithms. An irregular peak is marked if an interval between the marked peak and the following R peak is substantially different from a participant’s average R–R interval. Trained students considered each questionable peak. For example, they made sure that the marked peak is actually an R peak rather than a P or T wave. We followed manual editing guidelines according to MW Technologies procedures. The ECG signal was amplified by a gain of a 1,000, filtered with a hamming windowing function and a muscle noise filter. We derived time domain and frequency domain measures of HRV. The time domain measures we extracted were RMSSD, and SDNN. The frequency domain measure we derived was absolute high frequency (HF; 0.15–0.40 Hz) spectral power. These HRV measures were chosen since they represent both time and frequently domains, and since they seem to be the most common HRV measures considered in psychophysiological research (Blascovich et al., 2011). To look at specific frequencies of variability of IBI, the MW’s HRV application transforms the IBI series from the time domain to the frequency domain by Fast Fourier Transformation (FFT). The frequency of the interpolation is set to be 10 times the maximum acceptable HR that is defined as 200 BPM, which is the roughly maximum HR of young adult humans. For the results would be in the same measurement units as output by the E4, they were log transformed. For each one of the three segments (first baseline, conversation and second baseline) we looked for the raw IBI series, mean IBI, mean HR, RMSSD, SDNN (in milliseconds) and HF, all were extracted by the MW’s HRV application.

The analysis of the EDA signal obtained from the MW mobile device was performed using MW Technology’s EDA application software, version 3.1.5. Visual inspection and manual editing of the data were completed by a trained graduate student to ensure proper removal of artifacts. Manual visual inspection by trained students was performed in order to detect irregular sharp changes and sudden fluxes or drops in the data, which are clearly artifacts related to disconnections, and were correct by the spline function included in the application (which performs a linear interpolation on missing data). The EDA signal was smoothed with a rolling filter of 500 data points per block. We outputted the level of EDA in MicroSiemens (threshold of 0.05 as recommended by the provider) for every 500 ms. For each one of the three segments, we looked for the raw time-series of skin conductance level (SCL) and for the mean SCL.

#### Empatica E4 Wristband

The E4 wristband is equipped with sensors designed to gather high-quality data, such as photoplethysmogram sensor, from which HR, HRV, and IBI are derived through the BVP data. The photoplethysmogram sensor includes a light-source which illuminates the skin and a photo-detector which detects the intensity of the light refracted. The estimation of HR/IBI is based on the changes in the intensity of the refracted light caused by the fluctuations in blood flow (Tamura et al., 2014; Pietilä et al., 2017). The Empatica E4 outputs two main time-series for each individual, which give us insight into HR function: (1) HR and (2) IBI. Both are derived from the photoplethysmogram sensor of the Empatica E4. HR is estimated for every second via an Empatica E4 algorithm. IBI is presented as raw data collected. The HR spreadsheet contains one column, which represent HR sampled at 64 Hz (the default in the E4, and cannot be modify). The IBI spreadsheet includes two columns, one represents time since the E4 was operated in the beginning of the experiment (first row is the Unix time), and the second represents time in milliseconds between each successive heart-beats that were detected – after removing wrong beats by using a specific Empatica algorithm, so that the IBI represents the time in milliseconds between two successive heart-beats (R–R). Thus, as HR increases IBI decreases. The E4’s algorithm removes wrong beats when the difference between two successive beats is not in line with other intervals in the time-series. The timings of wrong beats are not included in the IBI spreadsheet. Therefore, it may happen that two consecutive rows in the IBI spreadsheet are not consistent with a standard tachogram. Thus, in the current study we also conducted visual inspection and manual editing of the raw IBI data.

After extraction of IBI data from the E4, it is recommended to use external software to derive HRV measures. Most of these software are MATLAB based (The MathWorks, Inc., Natick, MA, United States), such as Kubios HRV (Tarvainen et al., 2014), ARTiiFACT (Kaufmann et al., 2011), and KARDIA (Perakakis et al., 2010). R packages are also available, such as HRVR (García et al., 2012). We used Kubios HRV Premium analysis software version 3.1.0 (Tarvainen et al., 2014). We imported the raw data obtained from the E4 wristband into Kubios, after it was organized to fit Kubios’ specifications with MATLAB 2018a. Kubios HRV Premium software includes an adaptive QRS detection algorithm, pulse wave detectors and tools for automatic and threshold based artifact correction, trend removal and analysis sample selection (Tarvainen et al., 2014). We used the automatic correction method offered in Kubios (see specifications: Tarvainen et al., 2019).

Data analysis in Kubios Premium was performed by trained graduate students for the three segments collected (two baselines and the conversation) according to the 5-min events in the study’s procedure. For each 5-min segment we looked for the raw time-series of IBI, as well as the mean IBI, mean HR, RMSSD, SDNN, HF and the percent of data corrected by the software. For computing HF spectral power (0.15–0.40 Hz), we applied FFT just as we did in MW HRV. We chose to have 5-min segments following recommendations of the Task Force of The European Society of Cardiology and the North American Society for Pacing and Electrophysiology (Camm et al., 1996).

Electro-dermal activity is derived from two sensors that constantly measure fluctuating changes in certain electrical properties of the skin (Empatica, 2019). The EDA sensors are a unique feature to the E4 wristband, as other available commercial portable devices currently do not contain this capability. The output of the E4 includes a spreadsheet that contains one column in which SCL in MicroSiemens at 4 Hz sample is specified. Similarly to HRV, special software or code is needed to derive scaled, clean and meaningful EDA data – like Ledalab (free) or MindWare EDA application which we used in the current study. We used MW’s EDA application software, version 3.1.5. Visual inspection and manual editing of the data were completed by a trained graduate student to ensure proper removal of artifacts, just as we did with the EDA data collected by MW mobile device. The EDA signal was smoothed with a rolling filter of 500 data points per block. We outputted the level of EDA in MicroSiemens for every 500 ms.

The E4 also includes a 3-axis accelerometer, which captures motion-based activity and yields temporal information regarding acceleration in the space on *X*, *Y*, and *Z* axes. The XYZ raw acceleration is sampled by the E4 at 32 Hz and acceleration data point is outputted for each axis. Since data collected by a photoplethysmogram sensor is suspected to be particularly sensitive to movement (Tamura et al., 2014), which is an inherent aspect of interactive states (gestures during a conversation for example), we were also interested in the acceleration data on the three axes outputted by the Empatica E4. In order to investigate the impact of wrist movement on the accuracy of the E4 data, we calculated a proxy for movement on the *X*, *Y*, and *Z* axes. In order to calculate this proxy, we computed standard deviations (STDs) for each axis’s time-series. Note that for a completely static participant, the STD will be zero, and it will grow as the participant is more dynamic. We looked for movement proxy for each axis separately across the three conditions since we aimed to understand which directions of movement may be more frequently at play in the various conditions.

The E4 is also equipped with an infrared thermopile sensor which reads peripheral skin temperature and with an event mark button which allows participants to tag events which can be later linked to the different events occurred during the experiment (e.g., when a baseline starts or when the manipulation ends). In addition, the E4 contains an internal clock, a 5 ppm high accuracy time reference. The timestamp is set by the E4 manager when being connect to a PC via USB (Empatica, 2018).

To conclude, MW mobile device allows to collect IBI and HRV data by using electrode derived ECG, whereas IBI and HRV data gathered by the E4 is done with a photoplethysmogram sensor. Similarly, EDA data via the MW mobile device, is done by using two electrodes connected to the palm, whereas EDA data gathered by the E4 is done with their specialized EDA sensors.

### Procedure

Pairs of participants were invited to take part in a technology validation study. Upon arrival, they underwent informed consent and then they were asked to fill in a short demographic questionnaire which included items such as age and gender. In addition, they were asked to indicate the last time they ate and consumed caffeine, tobacco, and medicines. Individuals with known cardiac disease or pregnancy were excluded from the study. Following, each member of the dyad was connected to both the E4 wristband and to the MW mobile device by an experimenter: a same-gender undergraduate student previously trained to do so in our lab.

Each participant was instructed to wear the E4 wristband around the wrist of the non-dominant hand, according with the E4 manual - in a manner that it will be tight, but will not make him or her feel uncomfortable. Following the manual, participants were instructed to wear the wristband so it will be above the wrist joint and to line up the EDA sensors under the middle and ring fingers. If the participant needed help, the experimenters in charge assisted. Then, each member of the dyad was connected to one MW mobile device. The two MW mobile devices were connected wirelessly to a laptop computer in the control room adjacent to the lab room. The laptop acquired data from both MW mobile devices simultaneously.

After being connected to both the E4 wristband and to the MW mobile device, participants were asked to sit down and a 5-min baseline was taken, in which participants were asked to “sit still and relax.” As the baseline started, the experimenter left the room. After exactly 5 min the experimenter came back into the room, and instructed participants to stay in the same position and to have a 5-min conversation. During the conversation segment, participants were asked to discuss why they chose to enroll to the university, what they liked about it and what they disliked about it. We chose this topic since all participants were students and academic studies were a common theme for them. After 5 min, the conversation was stopped by the experimenter, and another 5-min of baseline was collected. As each segment of the experiment started and ended (baselines and conversation), participants were instructed to press the mark button located on the E4 wristband for 1 s. This was used to set a marker on the Empatica E4 data for the later analysis according to the E4 manual. When participants marked their wristbands, the experimenter also marked an event in the MW system’s laptop using BioLab Acquisition Software - MindWare’s acquisition and laboratory integration platform. After the second baseline ended, participants were thanked and disconnected from the electrodes and devices. The lab visit was videotaped from two angles allowing full visibility of both interacting partners’ faces and bodies.

### Examination of the Correlations Between the E4 Wristband and the MindWare Mobile Device

For each participant, for each one of the three segments of the experiment, and for each of the two continuous measures (IBI and EDA), we created a spreadsheet. In the spreadsheet, we positioned the two IBI time-series or the two EDA time-series, obtained by the E4 wristband and by the MW mobile device, side by side, making sure they are in the same length and on the same time-line. If not, the longer one was trimmed. This could happen when the participant did not press the mark button precisely on time, or when the E4 had missing data at the start or at the end of the segment (after being corrected. MW’s IBI series did not contain missing data). The trimming decisions were made after visual inspection of the data and in some cases after viewing the video recordings of the experiment. Then, we ran a cross correlation function (CCF) on the two time-series collected by the E4 wristband and the MW mobile device. We used R’s base package (R Core Team, 2013) to estimate the maximum correlation between the two time-series. This procedure enabled us to optimally align the two time-series derived from E4 and MW within a window of a few seconds and to assess the level of correlation between them. We also plotted the two series together on a scatter plot, making sure by visual inspection that any discrepancy was not related to time gaps.

## Results

### IBI

We first present mean IBI obtained from the E4 wristband and from the MW mobile device in each segment for each participant. We also present the percentage of data corrected by the Kubios HRV Premium software, when data obtained by the E4 contained missing, extra or ectopic beats (see Table 1).

**TABLE 1:** Mean IBI obtained by the E4 and by MW mobile device for each participant, in each of the three segments of the experiment, and percentage of data corrected by the external Software in the three segments.

Segment	First baseline				Conversation			Second baseline	
Participant	%E4’s corrected data	IBI		% E4’s corrected data	IBI		%E4’s corrected data	IBI	
		E4	MW		E4	MW		E4	MW
1	8.43	850	836	17.90	743	724	28.90	925	943
2	1.70	851	850	3.19	904	894	1.56	889	893
3	1.92	618	618	5.27	584	574	3.87	587	584
4	6.07	875	872	11.40	829	816	6.23	827	827
5	1.24	798	801	9.80	771	743	3.65	759	757
6	0.24	815	815	8.65	785	767	7.03	720	719
7	1.90	671	665	6.39	556	523	1.98	718	715
8	1.61	746	745	8.11	700	678	3.49	753	750
9	3.36	819	810	7.44	810	789	3.12	821	808
10	3.03	688	692	9.61	640	634	1.69	675	673
11	1.27	866	863	4.52	823	819	1.71	867	863
12	0.22	672	670	5.71	667	656	0.86	648	646
13	3.72	763	763	4.18	734	724	2.86	808	803
14	3.23	647	641	4.71	688	660	4.03	663	664
15	NA	NA	841	NA	NA	782	NA	NA	818
16	5.79	928	927	14.70	898	889	8.20	923	918
17	1.43	1054	1053	4.42	1058	1046	4.40	1096	1093
18	0.86	889	886	12.40	870	838	10.10	905	885
19	0.49	768	762	14.90	775	761	0.80	764	760
20	0.81	649	647	1.54	617	617	0.63	638	636
21	0.86	857	860	9.64	850	835	3.19	860	873
22	3.84	774	778	10.40	662	688	2.37	794	793
23	3.99	933	932	3.97	815	808	0.59	916	911
24	1.69	839	839	6.77	827	808	3.47	846	842
25	0.74	596	597	2.16	622	622	1.43	605	604
26	1.32	1006	1005	2.93	966	949	3.11	949	942
27	0.73	741	737	2.46	638	633	1.15	714	712
28	4.53	857	848	2.72	810	806	5.66	836	833
29	2.98	836	832	18.80	791	757	11.50	814	802
30	5.94	574	572	7.69	579	576	6.67	583	585
Mean^∗^	2.55	792	792	7.67	759	747	4.63	790	788

*IBI, interbeat interval in milliseconds; E4, Empatica E4 wristband; MW, MindWare mobile device. ^∗^Means do not include participant number 15 since his/her E4 data was unreliable.*

We could not extract reliable E4 IBI data from one participant (number 15) due to missing data (over 90%) and artifacts. One potential reason for this unusual amount of missing data might be the result of a wristband worn not tight enough or not in the exact location. This is despite the fact that we followed manufacturer guidelines when fitting the E4 to our participants. We decided to include participants with relatively high missing E4 data (more than 10% but less than 30%) since portable devices are characterized by relatively high missing data. We had one participant with relatively high RMSSD derived from MW mobile device (Zscore = 3.5), however, we decided to include this participant in the analysis after verifying that this relatively high score was not due to any measurement artifacts. As can be seen in Table 1, mean IBIs obtained by the E4 wristband and by the MW mobile device are highly similar, even when over one quarter of the data was corrected. Pearson correlation analysis revealed a very high correlation coefficient between mean IBIs obtained by the E4 and by the MW mobile device in the first baseline, *r*(27) = 0.999, *p* < 0.001, in the second baseline, *r*(27) = 0.998, *p* < 0.001, and in the conversation segment, *r*(27) = 0.995, *p* < 0.001. Similarly, we found nearly perfect correlations between mean HR obtained by the E4 and by the MW mobile device (see Table 2). As for the HRV parasympathetic measures mean RMSSD and mean HF, the results are mixed. For both measures, correlations are medium–high (0.74–0.88) during the baselines, but lower (0.42 and 0.46) during the conversation (see Table 2). As for mean SDNN, results are again mixed, with higher correlation during baselines (0.85 and 0.84) than during the conversation (0.67).

**TABLE 2:** Mean (standard deviation) IBI, HR, RMSSD, SDNDD, and HF in each segment obtained by both the E4 and by the MW mobile device, and the correlation between the means obtained by the two devices.

Measure	First baseline		Correlation coefficient (*r*)	Conversation		Correlation coefficient (*r*)	Second baseline		Correlation coefficient (*r*)
	E4	MW		E4	MW		E4	MW	
IBI	792 (117)	792 (117)	0.999^∗∗∗^	759 (119)	747 (118)	0.995^∗∗∗^	790 (121)	788 (120)	0.999^∗∗∗^
HR	77.64 (12.22)	77.68 (11.99)	0.997^∗∗∗^	81.07 (12.92)	82.48 (13.22)	0.992^∗∗∗^	77.90 (12.16)	78.18 (12.42)	0.999^∗∗∗^
RMSSD	52.97 (18.86)	41.59 (23.93)	0.743^∗∗∗^	61.71 (17.03)	42.00 (23.76)	0.417^∗^	55.38 (23.81)	39.36 (22.92)	0.812^∗∗∗^
SDNN	48.58 (13.79)	56.84 (17.35)	0.854^∗∗∗^	55.22 (11.23)	72.51 (18.70)	0.667^∗∗∗^	49.67 (15.80)	56.81 (19.72)	0.844^∗∗∗^
HF	6.41 (0.79)	5.96 (0.88)	0.885^∗∗∗^	6.20 (0.63)	6.06 (0.82)	0.462^∗^	6.24 (1.01)	6.00 (0.85)	0.781^∗∗∗^

**N* = 29; IBI, interbeat interval in milliseconds; HR, heart rate; RMSSD, Root Mean Square of Successive Differences; SDNN, Standard Deviation of Normal-to-Normal; HF, high frequency spectral power; E4, Empatica E4 wristband; MW, MindWare mobile device. ^∗^*p* < 0.05, ^∗∗∗^*p* ≤ 0.001.*

As for the change between situations, the changes captured by both devices seem to be more accurate for IBI and HR in comparison to HRV measures (see Table 3). For example, the E4 indicates a change of 4.42% in HR between baseline and conversation segments, while MW device indicates a change of 6.18% in HR between these situations. As for HRV measures, there was relatively big discrepancies between devices in detecting changes between situations. For example, the E4 indicates a change of 16.50% in RMSSD between baseline and conversations segments, whereas the MW device indicates a change of only 0.99% in RMSSD between these situations.

**TABLE 3:** Change in percent in IBI, HR, RMSSD, SDNN, and HF between situations, captured by both devices.

Measure	% Change from baseline to conversation		% Change from conversation to second baseline	
	E4	MW	E4	MW
IBI	–4.17	–5.68	4.08	5.59
HR	4.42	6.18	–3.91	–5.21
RMSSD	16.50	0.99	–10.26	–6.29
SDNN	13.36	28.01	–10.04	–21.66
HF	–3.35	1.62	0.57	–1.07

**N* = 29; IBI, interbeat interval in milliseconds; HR, heart rate; RMSSD, Root Mean Square of Successive Differences; SDNN, Standard Deviation of Normal-to-Normal; HF, high frequency spectral power; E4, Empatica E4 wristband; MW, MindWare mobile device.*

### Time-Series Correlations

We further examined correlation between the entire IBIs time-series, obtained by both the E4 and the MW mobile device, for each participant. This analysis yielded a correlation coefficient for each participant for each segment of the experiment. We used CCF which allowed us to examine the maximum correlation between the two time-series with some lags. On average, the number of lags was 1.03 in the first baseline, 4.90 lags in the conversation and 6.61 lags in the second baseline. Note that each lag represents one heart-beat. The correlations that were found are presented in Table 4.

**TABLE 4:** Intra-participant correlations between IBI time-series obtained by the E4 and by the MW mobile device, in each of the three segments.

Participant	First baseline	Conversation	Second baseline
1	0.431	0.705	NA
2	0.942	0.929	0.953
3	0.906	0.842	0.909
4	0.771	0.676	0.651
5	0.850	0.898	0.841
6	0.987	0.816	0.380
7	0.738	0.918	0.906
8	0.935	0.780	0.812
9	0.887	0.843	0.971
10	0.963	0.807	0.809
11	0.957	0.936	0.965
12	0.948	0.908	0.938
13	0.908	0.941	0.900
14	0.806	0.780	0.731
15	NA	NA	NA
16	0.879	0.692	0.743
17	0.826	0.958	0.865
18	0.992	0.943	0.914
19	0.966	0.750	0.953
20	0.979	0.961	0.968
21	0.991	0.813	0.960
22	0.909	0.948	0.982
23	0.931	0.875	0.890
24	0.830	0.822	0.688
25	0.964	0.918	0.909
26	0.977	0.889	0.892
27	0.889	0.831	0.892
28	0.979	0.936	0.974
29	0.893	0.774	0.887
30	0.745	0.804	0.751
Mean	0.889	0.851	0.858

The mean correlations between the two devices was relatively high, in all three segments (0.851–0.889) as can be seen in Table 4.

**FIGURE 1:**
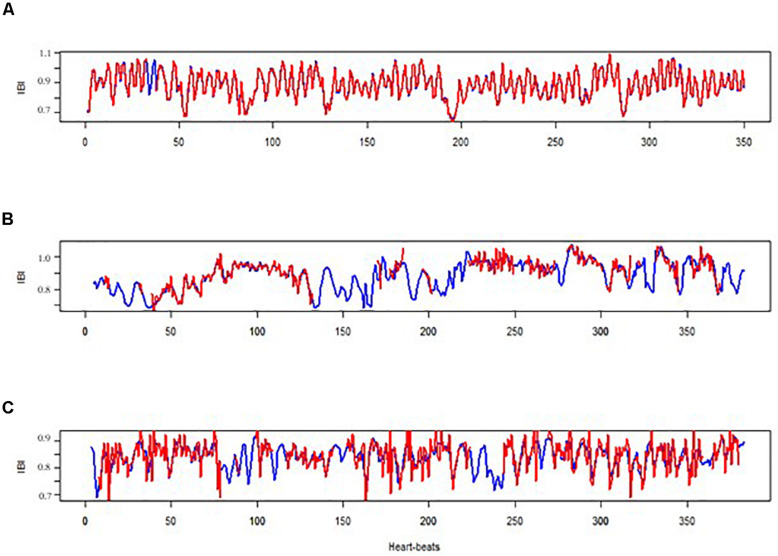
Three examples of two IBI time-series obtained by the E4 (in red) and by the MW mobile device (in blue). **(A)** Participant no. 18 during the first baseline; **(B)** participant no. 9 during the conversation; **(C)** participant no. 24 during the second baseline.

Figure 1 presents three examples of IBI time-series derived from the E4 and from MW in the same individual. The first example (Figure 1A) presents two highly similar time-series (*r* = 0.992) when the E4 time-series required just a small amount of data correction due to missing or poor data (0.86%). The second pair of IBI time-series (Figure 1B) is a more representative pair of time-series, with a medium–high correlation (*r* = 0.843) and an average conversation segment data correction due to missing or poor data in the E4 (7.44%). The third pair (Figure 1C) represents a below average correlation (*r* = 0.688) with a similar data correction to the average in the second baseline (3.47%).

The IBI series obtained by the E4 wristband had some missing data, which the Kubios HRV Premium software corrected by using its specified algorithm. There was significantly more corrected data during the conversation (7.67% of the data) compared to the first (2.55%) and second (4.63%) baselines. One-way repeated measures ANOVA revealed that that these differences are statistically significant, *F*(2,56) = 20.18, *p* < 0.001, η^2^ = 0.42. Pairwise comparisons revealed significant differences between the first baseline and the conversation, *p* < 0.001, and between the second baseline and the conversation, *p* = 0.002. There was no significant difference between the first and the second baseline, *p* = 0.054.

Next, we tested whether the amount of missing IBI data and artifacts from the E4 (which was later corrected for by the specialized Kubios HRV Premium software) accounted for the differences between measures derived from the E4 versus the MW mobile device. We computed the differences between the mean IBI obtained for each participant by the E4 and by the MW mobile device in absolute values, for each one of the three experimental segments. We then ran a Pearson correlation between the new delta variable computed and the amount of corrected IBI data from the E4. Results revealed a significant positive correlation *r*(85) = 0.60, *p* < 0.001. This means that as more data needed to be corrected, there was a higher level of difference between mean IBI obtained by the E4 the MW mobile device. Further, we speculated that one main reason for the amount of missing and poor data is the amount of wrist movement inherent to conversation compared to baselines. To examine this, we looked for correlations between the average movement of the participant on the *X*, *Y*, and *Z* axes, and the amount of data that was corrected for by the Kubios HRV Premium software, in each segment. Table 5 presents correlations between average movement proxy and corrected data in the three segments of the experiment.

**TABLE 5:** Mean (standard deviation) corrected data by the external software, mean movement on the *X*, *Y*, and *Z* axes and the correlation between them.

	Mean corrected data	Mean X movement	Correlation coefficient (*r*)	Mean Y movement	Correlation coefficient (*r*)	Mean Z movement	Correlation coefficient (*r*)
First baseline	2.55% (2.08%)	5.02 (4.63)	0.15	3.46 (4.48)	0.01	5.25 (9.74)	0.22
Conversation	7.67% (4.66%)	6.93 (5.64)	0.60^∗∗^	3.62 (4.03)	0.57^∗∗^	7.43 (10.18)	0.61^∗∗∗^
Second baseline	4.63% (5.34%)	5.41 (8.07)	0.01	2.58 (5.71)	0.24	5.34 (11.11)	–0.01

**N* = 29; ^∗∗^*p* < 0.01, ^∗∗∗^*p* < 0.001. X, movement on the *X*-axis; Y, movement on the *Y*-axis; Z, movement on the *Z*-axis.*

As can be seen in Table 5, we found a positive correlation between amount of corrected data and mean movement on the *X*, *Y*, and *Z* axes, but only in the conversation segment, when wrist movement was more prominent. There were no major differences between the axes with regard to correlations between movement and the amount of corrected data (see Table 5).

### EDA

In Table 6, we present the mean SCL obtained by both the E4 and the MW mobile device at each of the three experimental segments for each participant, after both series were cleaned by using MW EDA application.

**TABLE 6:** Mean skin conductance level in Microsiemens obtained by the E4 and by MW mobile device for each participant, in each of the three segments of the experiment.

	First baseline		Conversation		Second baseline	
Participant	E4	MW	E4	MW	E4	MW
1	0.14^∗^	0.55	0.14^∗^	0.75	0.14^∗^	0.51
2	1.07	10.70	1.86	12.72	2.53	14.05
3	0.45	0.88	0.94	5.24	0.88	1.63
4	0.24^∗^	3.37	0.22^∗^	9.21	0.20^∗^	5.30
5	1.67	16.82	1.15	18.56	0.84	18.12
6	0.15^∗^	3.19	0.16^∗^	11.71	0.18^∗^	9.83
7	0.37	3.57	0.65	10.87	0.23	4.54
8	0.29	9.54	0.56	13.44	1.05	13.09
9	0.26^∗^	10.16	0.23^∗^	13.16	0.21^∗^	11.47
10	0.24^∗^	14.66	0.27^∗^	17.07	0.30^∗^	11.84
11	0.45^∗^	10.53	0.43^∗^	13.28	0.46^∗^	11.51
12	0.21^∗^	9.81	0.23^∗^	13.16	0.25^∗^	11.17
13	0.20^∗^	7.87	0.19^∗^	12.04	0.21^∗^	11.08
14	1.72	9.43	3.61	11.80	3.90	11.25
15	0.22^∗^	1.45	0.26^∗^	1.43	0.29^∗^	1.41
16	0.22^∗^	13.49	0.26^∗^	24.91	0.29^∗^	19.97
17	0.15^∗^	0.63	0.22^∗^	2.09	0.28^∗^	2.64
18	0.33^∗^	2.53	0.21^∗^	5.51	0.16^∗^	2.68
19	0.32^∗^	12.75	0.36^∗^	24.79	0.36^∗^	15.22
20	0.23^∗^	7.91	0.42^∗^	11.92	0.23^∗^	10.47
21	0.12^∗^	3.92	0.14^∗^	9.65	0.17^∗^	6.98
22	0.17^∗^	4.30	0.21^∗^	7.30	0.24^∗^	6.85
23	0.11^∗^	1.19	0.11^∗^	1.33	0.12^∗^	1.36
24	0.30	5.84	0.99	11.41	1.08	8.69
25	0.22^∗^	11.19	0.23^∗^	17.89	0.17^∗^	13.88
26	0.22^∗^	1.70	0.20^∗^	3.09	0.19^∗^	2.91
27	3.39	20.16	7.03	20.22	8.17	19.01
28	0.30^∗^	9.62	0.24^∗^	9.53	0.34^∗^	11.20
29	0.08^∗^	4.71	0.09^∗^	7.85	0.10^∗^	8.89
30	0.48^∗^	10.87	0.13^∗^	12.56	0.18^∗^	12.61
Mean	0.48	7.68	0.73	11.15	0.79	9.34

*^∗^Data which may be strictly noise as the mean skin conductance level is close to zero and the EDA line is mostly flat. E4, Empatica E4 wristband; MW, MindWare mobile device.*

On average, we found a medium correlation between the EDA data (SCL) obtained by the E4 and by the MW mobile device in the first baseline, *r*(28) = 0.606, *p* < 0.001, and a relatively weak correlation in the second baseline, *r*(28) = 0.399, *p* = 0.03. We did not find a correlation between the mean SCL obtained by the E4 and by the MW mobile device during the conversation, *r*(28) = 0.298, *p* = 0.11. Flat lines around 0 μS and under 0.5 μS might imply that the recorded data was mostly noise (Empatica, 2015). That was the case for as many as 73% of the EDA data obtained by the E4 wristband, as a visual examination revealed.

**FIGURE 2:**
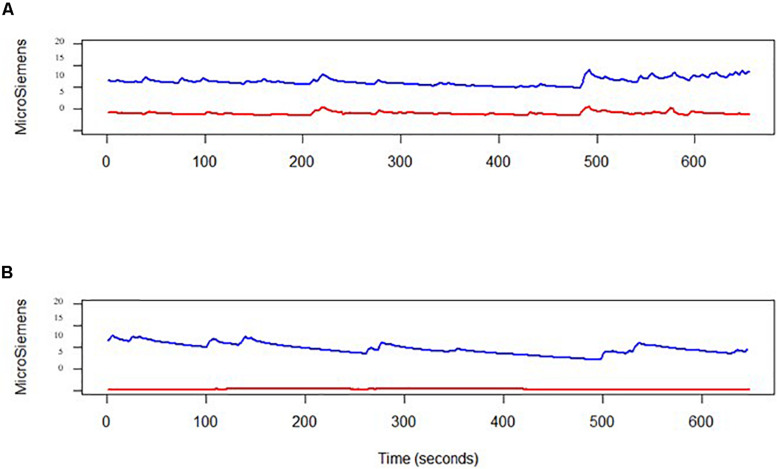
Two examples of two skin conductance level time-series obtained by both the E4 (in red) and by the MW mobile device (in blue). **(A)** Medium–high correlation (*r* = 0.538); **(B)** non-reliable E4 data collection, that was dropped from the analysis.

We did not look for intra-participant correlations between SCL time-series, or compared between other EDA measures, such as tonic period, since most of the data was not reliable. However, we present two examples in Figure 2. The first example presents a reliable data of participant number 14 during the second baseline, with a medium–high correlation (*r* = 0.538) between the E4 wristband and the MW mobile device. The bottom figure presents a non-reliable E4 SCL time-series of participant number 28 during the second baseline.

## Discussion

The current study asked to evaluate the quality of data derived from the Empatica E4 wristband specifically during naturalistic social interactions like a dyadic conversation, against the established MW mobile impedance cardiograph device. We found that the E4 wristband is a reliable tool for acquiring mean IBI and mean HR data. Further, after manual editing, the IBI time-series, derived from the photoplethysmogram sensor of the E4, was similar to the IBI series derived from the ECG data obtained by the MW mobile device via electrodes connected to the torso. We also found that the E4 was less accurate for HRV, obtained by Kubios Premium external software, and that it failed to produce reliable EDA data in our sample. These findings are in line with our hypotheses and with few other preliminary reports (McCarthy et al., 2016; Ollander et al., 2017; Pietilä et al., 2017; Ragot et al., 2018; Menghini et al., 2019).

The discrepancy in HRV measures between the two devices (with respect to absolute means and low correlations), especially in the conversation segment (in which participants were more dynamic) may be the results of different acquisition technologies. The E4 wristband acquires IBI by a photoplethysmogram sensor, which is based on BVP technology, and known to be sensitive to movement (Tamura et al., 2014), whereas the MW mobile device obtains the direct electrical ECG signal from electrodes, which is less sensitive to movement. In our study, the E4 cardiac output included relatively high missing data. In 10 out of the 90 measurements (30 participants, 3 segments) above 10% of the IBI data was missing. Some HRV measures are very sensitive to missing data, thus, the amount of missing data may certainly be a cause for the discrepancy between the devices with respect to HRV, especially when participants were more dynamic. Moreover, the two devices collect data at different sampling rates. MW mobile device collects ECG data at 500 Hz, whereas the E4 collects BVP data at 64 Hz, while the recommendation for reliable HRV measures are above 250 Hz for frequency domain measures and above 100 Hz for time domain measures (Kwon et al., 2018). However, despite the different technologies used to obtain and analyze the cardiac outputs, and the different sampling rates, both devices produced highly similar HR and IBI data (with respect to absolute means and correlations between time-series) in all three segments.

The E4 is thus recommended for those who are interested in mean HR/IBI or HR/IBI time-series during social interactions such as conversation. The first advantage of the E4 is that it is a non-invasive, portable, easy to use and light-weight device, which can be worn as a wristband or a watch. Thus, the E4 holds much less restrictions to the natural movement of the participant and allows for naturalistic settings. The second advantage is that it can be easily deployed outside the lab. With high duration battery (48+ h according to the provider; Empatica, 2019) and with an ability to work offline and to upload information to a cloud later on, it is very easy to use in field experiments, which can last up to 2 days.

Considering our results, we suggest that the E4 can be suitable for psychotherapy research focused on IBIs and specific HRV measures. For example, a growing body of research is focusing on client-therapist congruence (e.g., Stratford et al., 2012; Bar-Kalifa et al., 2019). The wearable device should not interrupt to the session and allow collecting physiological data during therapy, and calculating therapist-client physiological synchrony. Another setting in which the E4 might be of interest is the research regarding physiological synchrony. In recent years, there is a growing body of research that examines behavioral (Mogan et al., 2017) and physiological (Palumbo et al., 2017) synchrony in dyads or in groups. A great deal of this literature is looking for IBI or HR synchrony, when investigating dyadic or groups dynamics. Since we showed here that the IBI time-series yielded by the E4 is usually highly accurate, researches should consider using this device when investigating autonomic nervous system peripheral synchrony in dyads or in groups. The E4 might facilitate investigating group physiological synchrony in IBI and in HR, because of its simplicity and mobility. Scholars are calling for group physiological synchrony research, since groups are inherent part of our life, during the entire life span (Gordon et al., 2014). Yet, physiological group synchrony studies are scarce. We hope that by using the E4, this sort of research will be more prominent. We also suggest that the E4 may be more suitable for populations that will not be able or willing to wear multiple electrodes on their body, such as children and people with certain diagnoses. For example, Taj-Eldin et al. (2018) claim that wrist-worn devices are suitable for individuals with autistic spectrum disorders (ASD), while obtrusive devices, such as chest-worn devices, are the least suitable for people with ASD and intellectual disability. However, research should note that the E4 also contains some disadvantages.

Two great disadvantageous of the E4 is it is failure to produce reliable EDA data and the amount of missing IBI data, especially when the participant is being more dynamic. The external software remarkably succeeded to fill in the missing data and compute highly accurate IBI and HR data, as well as capturing changes between situations with respect to these measures. However, it had less success in computing HRV measures. There was a relatively high discrepancy between the means of the HRV measures, and correlations between the means were medium, especially when participants were dynamic. The E4 is able to compute highly accurate mean IBI and mean HR despite relatively high amount of missing data, when overcoming the missing, extra or ectopic beats - by the E4’s and by an external software’s algorithms. However, the E4’s algorithm and the use of an external software do not yield a highly accurate HRV data - both time and frequency domain. Thus, the E4 is recommended for those who are interested in proxy measures of HRV, especially if the procedure does not require much movement.

We found significantly more wrist movement and corrected data (implying missing and/or poor data) during the conversation, in comparison to the two baselines. Further, we found a positive correlation between wrist movement and the amount of corrected data in the conversation segment. This finding confirms our hypothesis that the photoplethysmogram sensor of the E4 (which is based on BVP technology) is sensitive to movement, and it is in-line with previous notions (see Tamura et al., 2014). We did not find a similar correlation in the two baselines, perhaps because there was almost no movement in this segment, as we asked the participants to sit still. Taken together, these results are specifically important for researchers aiming to use the E4 in dyadic research and collect data during naturalistic conversations. Considering that there is an immense interest in assessing naturalistic dyadic behavior as it occurs from a social neuroscience perspective (Zaki and Ochsner, 2009; Shamay-Tsoory and Mendelsohn, 2019), it is extremely informing to know in advance what the E4 can allow for in this context and what the limitation on posture and movement may be. We suggest that the optimal measure to assess via the E4 during naturalistic conversation is the mean IBI and mean HR and that participants in a study should be seated and asked to limit their arm movements if possible, and wear the E4 on their non-dominant hand.

Currently, it seems that there is no wristband which is capable of measuring both IBI data and EDA data accurately during naturalistic conversation settings. Competing devices, such as the Shimmer3 GSR + unit (Shimmer, Dublin, Ireland), allow collection of EDA data by a wristband device, however it includes two electrodes attached to the wristband and to two fingers, which might hold some restrictions to the natural movement of the participant. Similarly, the previous version of the E4, the E3, had been demonstrated as a highly accurate EDA measuring device, when two fingers were connected by electrodes to the wristband (Empatica, 2017). It may be, that for scientific purposes, the current options for a wrist measure of EDA is not satisfactory.

### Limitations and Suggestions for Future Research

The present study focuses on IBI, HRV, and EDA data during dyadic interactive states. Further research is needed to examine the validity of additional measures assessed by the Empatica E4 – acceleration and body temperature.

Some researchers point out advantages in measuring HRV data during a 24 h period (see Furlan et al., 1990; Malliani et al., 1994), specifically with respect to time-domain measures such as RMSSD and SDNN, while we measured HRV in three 5-min segments. However, most psychophysiological research does not measure HRV for 24 h, but for shorter segments. Also, a correlation between 5 min time-series might be a good indicator for longer time-series correlation, with less autocorrelation. One limitation of the current study’s design is that our control baseline condition did not include a “monolog” procedure which may account for speech artifacts. Future studies that focus on conversations should aim add a “monolog” baseline to the “rest” baseline condition to get a richer understanding of the impact of speech on psychophysiological measures.

In the current study, we used the same software (MW’s EDA application) to analyze EDA data extracted by both hardware (MW mobile device and the E4). However, this is not what we did for HRV data as the raw IBI series and the BVP data extracted by the E4 contain gaps in the data that MW’s HRV application cannot process as opposed to Kubios Premium, which we used here. Thus, HRV data obtained by the two devices had to be extracted from different software, which may account for some of the discrepancies between data outputted from the E4 compared to MW. However, a previous investigation provided evidence that both MW’s HRV application and Kubios produce highly similar results (Jarrin et al., 2012).

There is a debate in the literature whether linear analysis, as those we used in the current study, should be applied to HRV analysis (e.g., Kamath et al., 2012). However, as for IBI series, which we examined in the current investigation, there seems to be an agreement that linear methods are acceptable. For example Thuraisingham (2006) states that after removing ectopic beats and outliers by external software, linear analysis can be applied. Tom Kuusela concluded that “…typical time series as the sequence of R–R intervals, we can find in the literature a growing number of results that indicate this system to be stochastic rather than chaotic” (Kuusela, 2012, p. 10). Indeed, many researchers are performing Pearson correlations when examining relations between IBI/heart-rate series in validation studies (Barreira et al., 2009; Gregoski et al., 2012; Jarrin et al., 2012; Maheshkumar et al., 2016; Menghini et al., 2019). As for MEAN HRV measures, Pearson correlation’s assumptions are met and considered not only acceptable and common, but also adequate for examining relationships (rather than raw time-series).

We concluded that the E4 is a recommended tool for naturalistic studies that involve social conversations, if average IBI or HR scores are at interest. However, we conducted a lab experiment and not a field experiment, where we asked participants to have a conversation while seating. We were able to demonstrate that the E4 is a reliable tool for acquiring mean IBI and mean HR data during social interactions that included wrist movement. There is no reason to believe that the E4 will be less accurate outside the lab. However, if there is increased movement in a field experiment, for example during a conversation that occurs while walking together, we are not able to estimate how the E4 will fair. Future research should validate the E4 in the field when participants engage in conversations while standing up or performing daily activities such as driving, walking, or working.

## Data Availability Statement

The raw data supporting the conclusions of this article will be made available by the authors, without undue reservation.

## Ethics Statement

The studies involving human participants were reviewed and approved by Bar-Ilan University’s Department of Psychology’s IRB. The patients/participants provided their written informed consent to participate in this study.

## Author Contributions

IG conceived and designed the experiments. NM collected and analyzed the data, and prepared the figures and tables. NM wrote the main text of the study and IG provided edits and critical reviews. All authors contributed to the article and approved the submitted version.

## Conflict of Interest

The authors declare that the research was conducted in the absence of any commercial or financial relationships that could be construed as a potential conflict of interest.
